# Synergistic effects of multifactor interactions on the transmission of *Echinococcus* spp. on the Qinghai–Tibet Plateau

**DOI:** 10.1371/journal.pntd.0013985

**Published:** 2026-02-19

**Authors:** Ao Chen, Zhi Li, Ru Meng, Hongrun Ge, Xueyong Zhang, Hong Duo, Yuting Zhao, Zhihong Guo, Xiuying Shen, Rui Zhou, Yong Fu

**Affiliations:** 1 Academy of Animal Science and Veterinary, Qinghai Provincial Key Laboratory of Pathogen Diagnosis for Animal Diseases and Green Technical Research for Prevention and Control, College of Agriculture and Husbandry, State Key Laboratory for Diagnosis and Treatment of Severe Zoonotic Infectious Diseases, Key Laboratory for Zoonosis Research of the Ministry of Education, Qinghai University, Xining, China; 2 Xining Animal Disease Control Center, Xining, China; University of Zurich: Universitat Zurich, SWITZERLAND

## Abstract

The severe endemicity of *Echinococcus* spp. on the Qinghai‒Tibet Plateau (QTP) necessitates the identification of key risk factors influencing its transmission and distribution in northeastern QTP sylvatic cycles, alongside multifactorial interactions within the "environment–host–parasite" system. Field monitoring, multi source remote sensing data, and geographic detector techniques were integrated to elucidate the coupling relationships between the distribution and dissemination of *Echinococcus* spp. and geographical environmental factors. Land surface temperature (LST) was identified as a critical risk factor, exhibiting a significant negative correlation with *Echinococcus* spp. distribution (*P* < 0.01), while the interactive effects between factors surpassed individual impacts. The highest potential infection risk was localized in areas overlapping the northeastern plateau and the Three-River-Source core region, characterized by pronounced temperature fluctuations, low humidity, and intense radiation. On the basis of these findings, an ecoepidemiological hypothesis is proposed: the unique QTP habitat facilitates the evolution of a multi host parasitic system in *Echinococcus* spp.; subsequent host-mediated environmental modifications optimize dispersal conditions, jointly amplifying *Echinococcus* spp. transmission; and the synergistic coupling of "environmental-host-pathogen" dynamics underpins *Echinococcus* spp. endemicity on the QTP. This study provides a technical foundation for early risk warning and targeted control strategies for natural *Echinococcus* spp. foci.

## Author summary

In this study, we investigate how the environment of the Qinghai-Tibet Plateau influences the transmission of *Echinococcus* spp. By integrating field surveys with satellite data, we identify key environmental drivers such as land surface temperature, vegetation cover, and precipitation that shape the distribution of the parasite's wildlife hosts, including pikas, voles, wild foxes, stray dogs, etc. We demonstrate that the interaction between multiple factors, rather than any single variable, most strongly influences parasite spread. Our findings propose an ecoepidemiological framework linking plateau conditions, host adaptation, and pathogen persistence. This work provides a predictive tool for identifying high risk transmission zones and supports the development of targeted control strategies, contributing to management of zoonotic diseases in changing environments.

## Introduction

*Echinococcus* spp. are globally distributed, with nine currently recognized species. Cystic echinococcosis (CE) caused by *Echinococcus granulosus* has a worldwide prevalence, whereas alveolar echinococcosis (AE) induced by *E. multilocularis* has been documented exclusively in the Northern Hemisphere [1,2], both of which impose substantial burdens on animal husbandry and public health. No human infections caused by *E. shiquicus* have been reported, although its zoonotic potential cannot be excluded [3,4]. Predominantly, three species (*E. granulosus*, *E. multilocularis*, and *E. shiquicus*) are distributed across the Qinghai–Tibet Plateau (QTP) [5]. While *E. granulosus* and *E. multilocularis* are widely reported throughout China, *E. shiquicus* has thus far been detected only on the QTP [4]. An increasing trend in global echinococcosis incidence is projected for 2022–2035 [6]. As a hyperendemic region, the QTP exhibits sustained endemicity, which is postulated to be associated with its unique habitat and the abundance and distribution of suitable hosts. Further investigation into the interplay among environmental factors, host dynamics, and pathogen transmission is needed to inform control strategies of *Echinococcus* spp. on the QTP.

The transmission and distribution of *Echinococcus* spp. are influenced by multiple factors. Within sylvatic cycles, field survival rates of *Echinococcus* spp. eggs and population densities of primary hosts are considered pivotal [7,8], whereas in domestic cycles, pastoral sanitation conditions, livestock numbers, and stray dog populations are equally critical [9]. Environmental and biological determinants include documented correlations between the prevalence of *Echinococcus* spp. and factors such as altitude and land surface temperature [10,11]. The increased activity of definitive hosts, including *Vulpes ferrilata*, *Canis lupus laniger*, and *C. familiaris*, in suitable natural habitats facilitates extensive parasite dissemination [12–14], indicating close associations between these factors and *Echinococcus* spp. transmission and distribution on the QTP. Previous research has been precisely focused on single driving factors, yet most studies only address the individual effects of each factor, with the quantitative analysis of multi factor synergistic effects remaining insufficient [15–18]. Therefore, this study employs a geographical detector model to specifically investigate the mechanisms of multi factor interactions.

The QTP is characterized by a unique environment with an average altitude exceeding 4,000 m, dominated by alpine steppe and alpine meadow vegetation [19,20]. Persistent high radiation levels, low temperatures, aridity, and significant diurnal temperature fluctuations are documented on the QTP [21,22]. These habitats facilitate the extensive distribution of intermediate hosts of *Echinococcus* spp. (e.g., *Neodon fuscus* and *Ochotona curzoniae*), which exhibit heightened sensitivity to altitudinal and thermal gradients. Environmental factors, and the abundance and distribution of intermediate hosts are intrinsically linked to the population dynamics and spatial patterns of definitive hosts. Furthermore, complex topography, inadequate management of stray dogs, and anthropogenic disturbances significantly influence the transmission and distribution of *Echinococcus* spp. [23,24]. Consequently, mathematical modeling is needed to elucidate the interactions among environmental variables, host populations, and pathogen transmission, thereby providing a theoretical foundation for control strategies for *Echinococcus* spp. on the QTP.

Recent studies have indicated that environmental factors and disease distribution exhibit nonlinear relationships, necessitating nonlinear methodologies to elucidate the perturbing effects of multiple risk factors on the transmission and distribution of *Echinococcus* spp. [25,26]. To better interpret transmission dynamics on the QTP, in this study, a geographic detector model is employed to identify key risk factors influencing *Echinococcus* spp. epidemiology, and both individual and interactive effects of these factors are examined. Researches have demonstrated that interactive influences between factors generally exceed individual effects [27–29], as exemplified by anthrax where biological factors outweigh environmental determinants and interactions surpass singular impacts [30,31]. Consequently, this investigation incorporates plateau-specific environmental variables and *Echinococcus* spp. host related factors into a geographical detector model for comprehensive analysis.

In this study, field monitoring, multi source remote sensing, and geographic detection are integrated to elucidate the relationships between the distribution and transmission of *Echinococcus* spp. and geographical factors. The objectives include identifying environmental risk factors and spatial heterogeneities, thereby advancing the understanding of natural transmission mechanisms.

## Materials and methods

### Ethics statement

All animal experiments were performed in accordance with the guidelines of Institutional Animal Care and Use Committee of the Qinghai University (PJ202501–102).

### Investigation of the distribution and infection status of major hosts of *Echinococcus* spp. in the main natural foci on the Qinghai–Tibet plateau

Primary natural foci of *Echinococcus* spp. on the northeastern QTP were selected on the basis of the literature and historical epidemiological data. Systematic sampling was implemented across the study areas. Field sampling was conducted during the spring (April-May) and autumn (September-October) from 2021 to 2023. The population abundances of *N. fuscus*, *O. curzoniae*, *V. ferrilata*, and *C. familiaris* were quantified, The abundance of small mammal populations was quantified by sample square counting, with the sample square size being (100m × 100m). The population density of canines was estimated by the fecal square counting method (indirect counting method) (1km × 1km) [32]. A stratified proportional subset of *N. fuscus* and *O. curzoniae* was captured for dissection. Suspected *Echinococcus* spp.-infected viscera were preserved in 70% ethanol. Fecal samples from wild Canidae (*V. ferrilata*, *C. familiaris*, etc.) were stored at −70°C for molecular identification of host infection rates. For the molecular identification of *Echinococcus* spp. in fecal samples, PCR targeting the *cox1* gene was performed. The primers were synthesized by Sangon Biotech (Shanghai) Co., Ltd., with the forward primer (F): TTTTTTGGGCATCCTGAGGTTTAT and the reverse primer (R): TAAAGAAAGAACATAATGAAAATG [33].

### Extraction and selection of geographic environmental variables for the main natural foci on the Qinghai–Tibet plateau

During field investigations of primary *Echinococcus* spp. host distributions, handheld GPS units were used for site localization, with geographical coordinates recorded and environmental data collected onsite. Corresponding environmental variables were extracted and integrated with field-surveyed environmental data and *Echinococcus* spp. epidemiological findings for multifactorial analysis, enabling screening of eligible environmental variables. On the basis of prior research and survey results, 11 risk factors serving as environmental and biological proxies were incorporated into the geographic detector model, for detailed variable sources, resolutions and time ranges, please refer Table 1 [34]. All the meteorological and altitude data are sourced from open source websites and can be downloaded for free [35–37].

**Table 1:** The sources, resolutions and temporal range of 11 risk factor variables.

Variable (Abbr.)	Source & Description	Processed Spatial Resolution	Temporal Range
Radiation (RAD)	ERA5-Land: Monthly average of surface solar radiation downwards.	0.1° × 0.1°	Concurrent with host data monthly
Normalized difference vegetation index (NDVI)	MODIS MOD13A2 v6: Provides 1 km NDVI at 16-day composites.	1 km resampled	Concurrent with meteo data
Land surface temperature(LST)	ERA5-Land: Monthly average of skin temperature.	0.1° × 0.1°	Concurrent with host data monthly
Elevation (ELE)	China 1000m Resolution Digital Elevation Model	1km resampled	Static
Small mammal count (SMC)	Obtained through field surveys	Survey points	Spring/Autumn 2021-2023
Canidae count (CC)	Obtained through field surveys	Survey points	Spring/Autumn 2021-2023
Wind speed (WS)	ERA5-Land: Monthly average of 10m u and v-component of wind.	0.1° × 0.1°	Concurrent with host data monthly
Precipitation (PRE)	ERA5-Land: Monthly total of total precipitation.	0.1° × 0.1°	Concurrent with host data monthly
Relative humidity (RH)	ERA5-Land: Monthly average of 2m dewpoint temperature, used to calculate RH.	0.1° × 0.1°	Concurrent with host data monthly
Mean annual temperature(TEM)	ERA5-Land: Monthly average of 2m temperature.	0.1° × 0.1°	Concurrent with host data monthly
Sunshine hours (SH)	Calculated from ERA5-Land’s monthly solar radiation (RAD) using astronomical formulae.	0.1° × 0.1°	Concurrent with host data monthly

### Detection of geographic environmental risk factors for the natural foci of *Echinococcus* spp. on the QTP

The geographic detector method quantifies explanatory power by comparing variances between classified independent variables and dependent variables, comprising four modules: the factor detector, interaction detector, risk detector, and ecological detector [38]. The correlation between environmental risk factors and *Echinococcus* spp. was demonstrated by the Mantel test. The influence of environmental factors on the dynamics of the *Echinococcus* spp. host population was fitted with a generalized additive model (GAM). Both of these processes were run using code in R studio. The Mantel test was implemented with the "mantel_test()" function from the linkET package (version ≥ 0.0.3) to assess the correlation between species community matrices and environmental factor matrices. GAM were constructed using the gam() function from the mgcv package (version ≥ 1.9). The specific GAM formula used was: Host_Density ~ s(variables, k = 5, bs ='tp'), where "s()" denotes a smooth term, "k=5" sets the upper limit of basis dimension for the smooth to 5, and"bs='tp'" specifies the thin-plate regression spline as the smoother basis. Models were fitted using the Restricted Maximum Likelihood method. The Mantel test employed a Bray-Curtis distance matrix for species abundance and a Euclidean distance matrix for environmental factors, with significance determined via 999 permutations.

Factor detection: The *q*-statistic measures the spatial heterogeneity and explanatory power of environmental factors, where *q*∈[0, 1]. A relative high *q* indicates relatively strong influence on the distribution and transmission of *Echinococcus* spp.. The formula is as follows:


$$q=\text{1}-\frac{\sum_{i=\text{1}}^{n}N_{i}\sigma_{i}^{\text{2}}}{N\sigma^{\text{2}}}$$

In the formula, *q* represents the influence of a certain environmental factor on the distribution and spread of *Echinococcus* spp., *i* = 1, 2... n represents the geographical environmental division of the research area. *Ni* represents the number of environmental factors (including altitude, climate, precipitation, vegetation and other environmental factors in the *Echinococcus* spp. habitat) corresponding to zone *i*. N represents the total number of environmental factors in the study area; $\sigma_{i}^{\text{2}}$ and $\sigma^{\text{2}}$ represent the discrete variance of partition *i* and the entire partition, respectively.

Interactive detection: A comparison of the interactive effects of two environmental factors, A and B, in the *Echinococcus* spp. habitat on the distribution and spread of *Echinococcus* spp., can reveal whether they act independently or show a trend toward enhanced or weakened interaction on the distribution and spread of *Echinococcus* spp.. The specific comparison results and classification types are shown in Table 2.

**Table 2:** Classification of the results of the detection of interactions of dual environmental factors.

Criterion	Interaction relationship
*q*(A∩B) < Min (*q* (A), *q* (B))	Weaken, nonlinear-
Min(*q* (A), *q* (B)) < *q*(A∩B) < Max(*q*(A), *q*(B))	Weaken
*q*(A∩B) > Max (*q*(A), *q*(B))	Enhance, bi-
*q*(A∩B) = *q*(A) + *q*(B)	Independent
*q* (A∩B) > *q*(A) + *q*(B)	Enhance, nonlinear-

**Note**: *q* represents the influence of a certain environmental factor on the distribution and spread of *Echinococcus* spp.. A and B represent two different environmental factors in the habitat of *Echinococcus* spp..

Risk detector: By comparing the significant differences in the mean values of the environmental factors within each zone, the potential epidemic risk areas of the natural foci of *Echinococcus* spp. in the QTP were detected. The *t* statistic was used to represent the test formula as follows:


$$t_{{\overset{―}{y}}_{h=\text{1}}-{\overset{―}{y}}_{h=\text{2}}}=\frac{{\overset{―}{Y}}_{h=\text{1}}-{\overset{―}{Y}}_{h=\text{2}}}{{\left(\frac{Var({\overset{―}{Y}}_{h=\text{1}})}{n_{h=\text{1}}}+\frac{Var({\overset{―}{Y}}_{h=\text{2}})}{n_{h=\text{2}}}\right)}^{\text{1}/\text{2}}}$$

In the formula, ${\overset{―}{Y}}_{h}$ represents the average value of research attributes (such as the prevalence of *Echinococcus* spp.) in different zones; $n_{h\;}$ represents the number of environmental factors in different partitions; and *Var* represents the variance. The statistic *t* approximately follows Student’s *t* distribution. The null hypothesis *H*_0_ is as follows: ${\overset{―}{Y}}_{h=\text{1}}={\overset{―}{Y}}_{h=\text{2}}$. If *H*_0_ is rejec*t*ed at confidence level α, there is a significant difference in *t*he mean values of the study attributes between the two different partitions.

Ecological detector: The *F* test is used to measure whether there are significant differences in the influence of various environmental factors on the spatial distribution of *Echinococcus* spp.. The formula is as follows:


$$F=\frac{N_{X\text{1}}(N_{X\text{2}}-\text{1})\sum_{X\text{1}=\text{1}}^{n\text{1}}N_{X\text{1}}\sigma_{X\text{1}}^{\text{2}}}{N_{X\text{2}}(N_{X\text{1}}-\text{1})\sum_{X\text{2}=\text{1}}^{n\text{2}}N_{X\text{2}}\sigma_{X\text{2}}^{\text{2}}}$$

In the formula, $N_{X\;\text{1}}{,\;N}_{X\text{2}}$ represents the number of environmental factors *X*1 and *X*2 in the partitions of the study region. $\sum_{X\text{1}=\text{1}}^{n\text{1}}N_{X\text{1}}\sigma_{X\text{1}}^{\text{2}}\;$ and $\sum_{X\text{2}=\text{1}}^{n\text{2}}N_{X\text{2}}\sigma_{X\text{2}}^{\text{2}}$ represent the sum of the variances within the partition formed by *X*1 and *X*2, respectively. *n*1 and *n*2 represent the number of partitions for the environmental factor variables *X*1 and *X*2, respectively. Here, the null hypothesis *H*_0_ is as follows: $\sum_{X\text{1}=\text{1}}^{n\text{1}}N_{X\text{1}}\sigma_{X\text{1}}^{\text{2}}=\sum_{X\text{2}=\text{1}}^{n\text{2}}N_{X\text{2}}\sigma_{X\text{2}}^{\text{2}}$. If *H*_0_ is rejected at the significance level of α, there is a significant difference in the influence of the 2 environmental factors on the spatial distribution of *Echinococcus* spp..

## Results

### Spatial variation and basis of pathogenic ecology of *Echinococcus* spp. foci on the Northeastern Qinghai–Tibet Plateau

**Fig 1:**
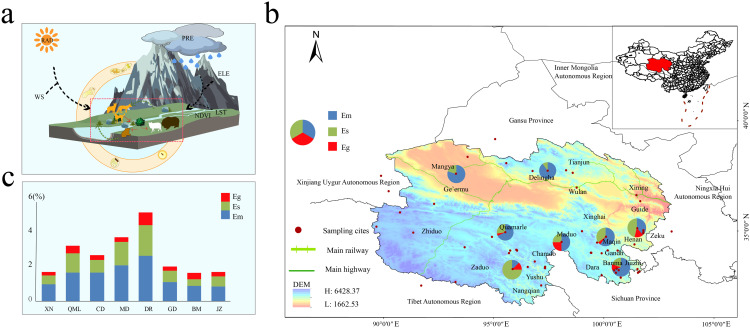
Spatial variation in *Echinococcus* spp. foci on the northeastern Qinghai–Tibet Plateau. a: "Host-environment-pathogen" interactions of *Echinococcus* spp. in the northeastern QTP environment. Environmental factors: LST (land surface temperature), ELE (elevation), NDVI (normalized difference vegetation index), PRE (precipitation), WS (wind speed), RAD (radiation), and biological factors: canines and small mammals. Both biological and environmental factors have impacts on stages of *Echinococcus* spp.. b: Spatial distribution of *Echinococcus* spp. foci and infection rates by county in the northeastern QTP. Combining historical data and sampling data, the sampling points and the approximate distribution of *Echinococcus* spp. were obtained. The base layer is from https://www.webmap.cn/mapDataAction.do?method=forw&datasfdafd=%253Fmethod%253Dforw%2526amp%253BresType%253D5%2526amp%253BstoreId%253D2%2526amp%253BstoreName%253D%2525E5%25259B%2525BD%2525E5%2525AE%2525B6%2525E5%25259F%2525BA%2525E7%2525A1%252580%2525E5%25259C%2525B0%2525E7%252590%252586%2525E4%2525BF%2525A1%2525E6%252581%2525AF%2525E4%2525B8%2525AD%2525E5%2525BF%252583%2526amp%253BfileId%253DBA420C422A254198BAA5ABAB9CAAFBC1 with credit to National Catalogue Service For Geographic Information. c: Infection rates in primary *Echinococcus* spp. foci in the northeastern QTP. XN: Xining, QML: Qumalai, CD: Chengduo, MD: Maduo, DR: Dari, GD: Gande, BM: Banma, JZ: Jiuzhi.

Fig 1a schematically illustrates the "environment–host–pathogen" interactions of *Echinococcus* spp. within the northeastern QTP habitat. The QTP environment is characterized predominantly by alpine meadows, steppes, and deserts, featuring low oxygen levels and arid conditions resulting in nutrient-poor soils. Wild Canidae (*V. ferrilata*, *C. familiaris*, etc.) are widely distributed, alongside abundant populations of small mammals (*O. curzoniae*, *N*. *fuscus*, etc.). Small mammals serve as crucial intermediate hosts for the sylvatic transmission of *Echinococcus* spp., and their population density significantly influences *Echinococcus* spp. infection rates. Field sampling and historical investigations revealed (Fig 1b) that *Echinococcus* spp. are widely distributed across the high altitude, hypoxic environment of the QTP. This unique habitat has led to the formation of a distinct survival pattern through long term adaptive evolution among the three enzootic *Echinococcus* spp. (*E. multilocularis*, *E. granulosus*, and *E. shiquicus*): *E. multilocularis* has the broadest geographical range, followed by *E. shiquicus*, with *E. granulosus* exhibiting the most restricted distribution. Statistical analysis of *Echinococcus* spp. infection rate data from major endemic foci in the northeastern QTP revealed that Dari and Maduo Counties presented the highest prevalence rates, whereas the lowest rate was observed in Xining city.

### Risk factor screening and the correlation between risk factors and *Echinococcus* spp.

**Fig 2:**
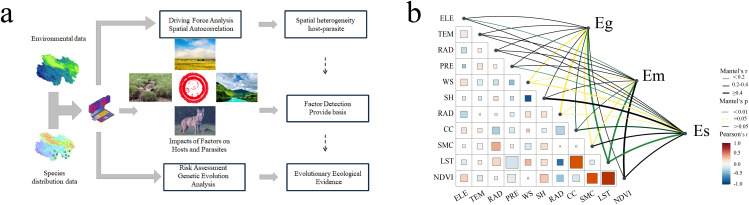
Screening of factors influencing the distribution of *Echinococcus* spp. in the northeastern QTP. a: Flowchart for screening influential factors. b: Mantel test matrix of species correlations between influential factors and three *Echinococcus* spp..

Fig 2a presents a technical flowchart that systematically illustrates the research pathway from screening factors influencing *Echinococcus* spp. distribution to analyzing "environment–host–pathogen" linkages. A Mantel test was then applied to quantify the correlation between the environmental factor matrix and the spatial distribution matrices of the three *Echinococcus* species (Fig 2b). The strength and direction of the associations between these eleven factors and the three *Echinococcus* species were visualized. LST was a significantly negatively correlated with Em, Eg, and Es (*P* < 0.01). The SMC exhibited a strong positive correlation with Em and Eg (*P* < 0.05). Conversely, RAD and WS were negatively correlated with all three species (*P* < 0.05).

### Optimal parameter-based geographic detector (OPGD) optimization and results of the detection of factors and interactions

To further elucidate the effects driving risk factors on *Echinococcus* spp. infection rates, a geographic detector model was employed for analysis of multifactor interactions. During factor preprocessing, the OPGD model was introduced to optimize the spatial discretization of continuous factors. For the 11 risk factors, four classification schemes, including the geometrical interval and quantile, were applied. The optimal number of classes was determined through iterative computation.

**Fig 3:**
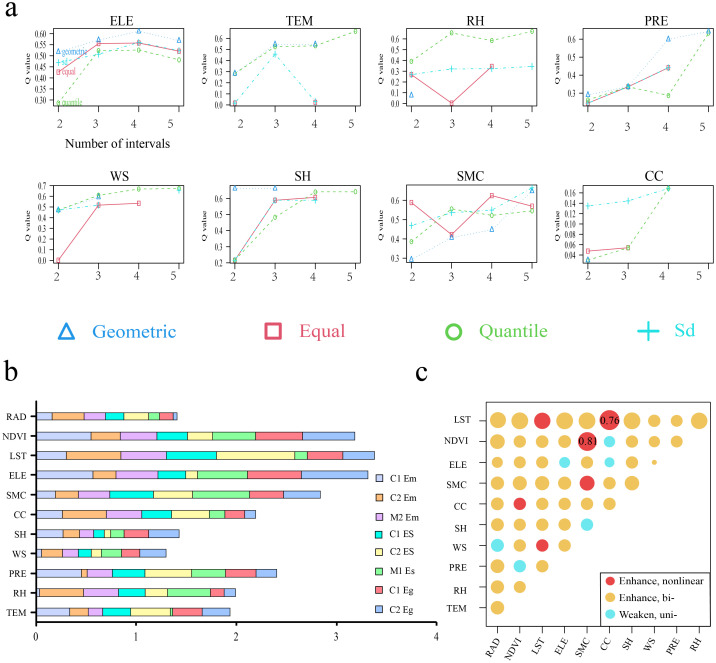
OPGD optimization and geographic detector analysis of risk factors influencing the transmission of *Echinococcus* spp.. a: OPGD optimization classification of selected risk factors for geographic detectors. The classification number represents the specific distribution of each factor in several intervals and is used to optimize the geographical detector. Select the category with the highest *q* value, or the one that arrives earliest and has a *q* value close to that. b: Results of the geographic detector and factor detection and *q* value rankings(C: Canines, C1: *Canis familiaris*, C2: *Vulpes ferrilata*; M: Small mammals, M1: *Ochotona curzoniae*, M2: *Microtus fuscus*). c: Results of the geographic detector and interaction detector.

As illustrated (Fig 3a), the *q* value for ELE peaked at 0.68 under a 4-class geometrical interval division, whereas the *q* value for the TEM approached its theoretical maximum (0.59) at 0.57 under a 3-class quantile division, leading to the adoption of three classes. Following OPGD screening, the factor detector results (Fig 3b) ranked the top 5 factors by explanatory power (*q* value) for *Echinococcus* spp. distribution as follows: LST, ELE, NDVI, SMC, and PRE. LST directly influenced the distribution range of primary hosts and egg development. ELE indirectly affected *Echinococcus* spp. transmission by shaping the vertical distribution of small mammals and Canidae. SMC, representing intermediate host abundance, directly determines the environmental pressure of infection sources. NDVI, indicating vegetation coverage, determines food availability and habitat suitability for intermediate hosts, while also regulating near ground climate, influencing egg survival. SMC represents intermediate host abundance, directly influences the infection intensity of *Echinococcus* spp. in sylvatic cycle. PRE indirectly regulates host distribution by affecting soil moisture and vegetation growth, and heavy rainfall may exert physical scouring on surface eggs. A higher *q* value in factor detector indicates a greater impact of the factor on the infection rate of *Echinococcus* spp.. The interaction detector results (Fig 3c) revealed that the intersection of NDVI∩SMC (*q* = 0.81) and LST∩CC (*q* = 0.76) had the strongest nonlinear enhancement effects on the distribution and transmission of *Echinococcus* spp.. Collectively, the results of the detection of factors and interactions demonstrate the synergistic effects of driving environmental and biological factors on the transmission and distribution of *Echinococcus* spp..

### Relationship between main environmental influencing factors and the dynamics of host populations

**Fig 4:**
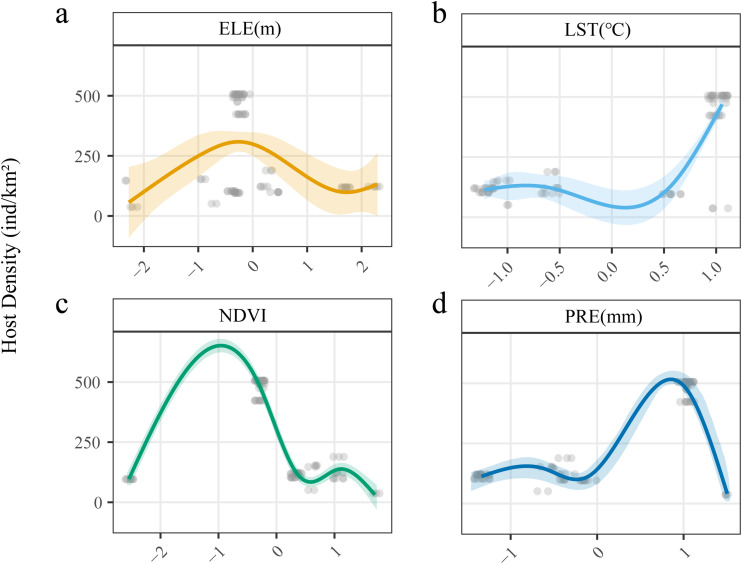
Influence of major environmental factors on primary hosts of *Echinococcus* spp. on the northeastern Qinghai–Tibet Plateau. The figure presents the key environmental drivers influencing the transmission and distribution of *Echinococcus* spp.. a: ELE Elevation. b: LST (Land surface temperature). c: NDVI (normalized difference vegetation index). d: PRE (precipitation). The colored area interval represents the confidence interval.

Fig 4 reveals the nonlinear regulatory effects of environmental factors on host population dynamics, demonstrating complex ecological responses between climatic variables and host density. As shown in Fig 4a, 4c, and 4d, unimodal curves characterize the relationships of ELE, NDVI, and PRE with host density peaking at intermediate environmental intensities, indicating optimal niche intervals for *Echinococcus* spp. hosts. A negative correlation with the LST is demonstrated (Fig 4b), suggesting that the LST influences the host spatial distribution and behavioral rhythms. Collectively, the GAM results confirm the existence of optimal ecological niches for *Echinococcus* spp. hosts, indicating potential maximum infection risk zones for *Echinococcus* spp..

### Potential risk assessment of *Echinococcus* spp. on the Northeastern QTP

**Fig 5:**
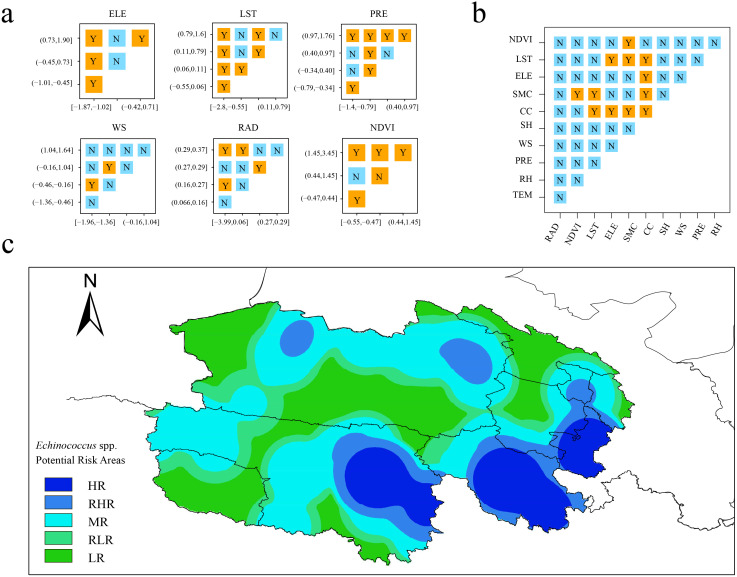
Redundancy analysis and risk assessment of environmental risk factors for *Echinococcus* spp. on the northeastern QTP. a: Results of the geographic detector and ecological detector. b: Results of the geographic detector and risk detector. c: Zonation of potential areas of *Echinococcus* spp. distribution on the northeastern QTP. Different colors represent the degree of potential infection risk of *Echinococcus* spp., HR: high risk, RHR: relatively high risk, MR: medium risk, RLR: relatively low risk, LR: low risk. The base layer is from https://www.webmap.cn/mapDataAction.do?method=forw&datasfdafd=%253Fmethod%253Dforw%2526amp%253BresType%253D5%2526amp%253BstoreId%253D2%2526amp%253BstoreName%253D%2525E5%25259B%2525BD%2525E5%2525AE%2525B6%2525E5%25259F%2525BA%2525E7%2525A1%252580%2525E5%25259C%2525B0%2525E7%252590%252586%2525E4%2525BF%2525A1%2525E6%252581%2525AF%2525E4%2525B8%2525AD%2525E5%2525BF%252583%2526amp%253BfileId%253DBA420C422A254198BAA5ABAB9CAAFBC1 with credit to National Catalogue Service For Geographic Information.

The results of the ecological detector (Fig 5a) determine whether there are structural differences in the overall distribution of the studied subject across different zones or types. Building on this, the risk detector (Fig 5b) further identifies which specific zones exhibit indicator values significantly above or below the average level, thereby precisely locating high risk and low risk areas. Fig 5c presents a zonation map for the potential distribution of *Echinococcus* spp. on the northeastern QTP. This map employs a five-tier classification system (HR–LR) to reveal spatial risk heterogeneity: high risk areas (dark blue) cluster in relatively high elevation regions, with Dari County (Golog Tibetan Autonomous Prefecture), Maduo County (Golog), and Chengduo County (Yushu Tibetan Autonomous Prefecture) exhibiting the highest infection risk. Low risk areas (light green, e.g., Gonghe County) correspond to lower altitude zones with intensive human activities.

## Discussion

In this study, the LST, ELE, NDVI, SMC, and PRE were identified as key risk factors influencing the transmission and distribution of *Echinococcus* spp. on the northeastern QTP. More importantly, the LST demonstrated the strongest negative correlation (*P* < 0.01), which is consistent with the findings of the previous studies [39]. Within sylvatic cycles, the LST governed spatial ranges of the hosts; infected hosts migrating to thermally suitable habitats facilitate transmission to new animals [40,41], with subsequent host relocations indirectly expanding the distribution of *Echinococcus* spp.. It is inferred here that high priority should be attached to climate change's future impact on *Echinococcus* spp. infection rates. First, the survival of eggs in current same altitude environments is prolonged and the development potentially accelerated by rising temperatures; second, altered host suitable ranges may expand transmission to higher altitudes; and third, indirect effects on host population dynamics are induced through altered NDVI and PRE patterns. Synergistic change of these factors could nonlinearly intensify the transmission of *Echinococcus* spp.. Unlike prior studies [34], this investigation incorporated wild canid and small mammal populations to examine "environmental‒host‒pathogen" interactions in sylvatic cycles. Canid abundance was excluded as a key predictor, potentially because of the substantially higher density of small mammals (53–278 individuals/ha for *O. curzoniae*) [42] and their elevated infection rates compared with those of canids. Small mammals also exhibited greater environmental modification capabilities than canids do [43,44], indirectly selecting parasite-favorable habitats. Geographic detector analysis revealed that the NDVI∩SMC (*q* = 0.81) and LST∩CC (*q* = 0.76) had the strongest synergistic effect on distribution, exceeding the impacts of individual factors and paralleling findings in leishmaniasis and emerging zoonoses [31,45,46]. We speculate that the interaction between environmental factors and hosts exerts a significant influence on the transmission and distribution of *Echinococcus* spp.. Appropriate land surface temperature and vegetation coverage lead to an extension of the predation range and duration for canines, alongside an expansion of the habitat and population size for small mammals, meanwhile, geographical conditions promote the development of eggs in the sylvatic cycle, and biological factors expand the distribution range of eggs. The unique QTP habitat and endemic species (e.g., *V. ferrilata*) are inferred to foster complex host‒environment interactions that limit single factor influences and emphasize multifactorial synergy. Environmental factors regulate both intermediate and definitive host distributions, compounded by trophic limitations within sylvatic cycles.

The QTP hosts extensive *Echinococcus* spp. host distributions, with key environmental factors influencing host population dynamics and defining optimal ecological niches for *Echinococcus* spp. in its northeastern region [20,29,47]. Risk and ecological detector analyses revealed the overlap between the northeastern QTP and the Three-River-Source core region as having the highest potential infection risk, characterized by substantial land surface temperature fluctuations, low humidity, and intense radiation. These findings align with recent epidemiological surveys [45,48,49]. Featuring predominantly alpine semiarid climates with low annual temperatures, these areas present elevated transmission risk due to frequent human and livestock contact with susceptible hosts, compounded by high stray dog concentrations near monasteries and settlements where effective deworming and canine management are challenging [50–52]. The potential distribution zoning map generated for the northeastern QTP provides a scientific basis for delineating endemic areas and implementing targeted control measures to mitigate parasite spread.

**Fig 6:**
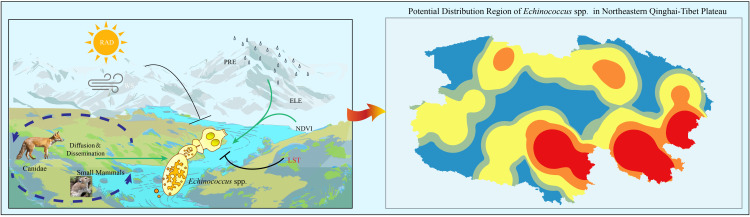
Mechanisms of the interplay of "environment–host–pathogen" factors for *Echinococcus* spp. on the northeastern QTP. The base layer is from https://www.webmap.cn/mapDataAction.do?method=forw&datasfdafd=%253Fmethod%253Dforw%2526amp%253BresType%253D5%2526amp%253BstoreId%253D2%2526amp%253BstoreName%253D%2525E5%25259B%2525BD%2525E5%2525AE%2525B6%2525E5%25259F%2525BA%2525E7%2525A1%252580%2525E5%25259C%2525B0%2525E7%252590%252586%2525E4%2525BF%2525A1%2525E6%252581%2525AF%2525E4%2525B8%2525AD%2525E5%2525BF%252583%2526amp%253BfileId%253DBA420C422A254198BAA5ABAB9CAAFBC1 with credit to National Catalogue Service For Geographic Information.

Some *Echinococcus* spp. lineages originated outside the QTP. Progressive QTP adaptation enabled expansion of the parasite's host range from *Ovis aries* to multiple species, reflecting pathogen adaptability in host selection and environmental acclimatization [53,54]. Miocene continental plate collision-induced QTP uplift drove environmental change, driving local host ecological divergence from East Asian ancestors [55–57]. Based on this, we propose a pathogen ecology hypothesis for the ecoepidemiological origin of *Echinococcus* spp. on the QTP (Fig 6): unique QTP habitat shaped the multihost system of *Echinococcus* spp.; host-mediated environmental modifications optimized dispersal conditions; and their interactions drove extensive *Echinococcus* spp. transmission. The coupled synergy among the environment, hosts, and pathogens constitutes the fundamental ecoepidemiological basis for the persistence of *Echinococcus* spp. on the QTP.

The synergistic effects identified here are comparably relevant to other high altitude, multi host parasite systems. In contrast to the broad host range of *Toxoplasma gondii*, *Echinococcus* spp. on the QTP exhibits pronounced dependence on specialized intermediate and definitive hosts within extreme environments, confining transmission to a more specialized ecological niche. Compared with system like leishmaniasis, the nonlinear interactions emphasized in this study are likely amplified by the unique conditions of QTP. However we acknowledge several limitations in the present study. The analysis was restricted to pairwise interactions, whereas the actual transmission of *Echinococcus* spp. involves complex co-occurrence of multiple factors [46,58]. Moreover, it is well established that *Echinococcus* spp. infection are associated not only with environmental and host factors but also influenced by human and social determinants. Previous studies have identified key risk factors in endemic areas, including low education levels, contaminated water sources, and engagement in work related to animal husbandry, with Kern P et al. particularly emphasizing the critical role of anthropogenic factors such as hygiene practices and canine management [59–62]. Furthermore, the model relies on an assumption of equilibrium conditions, potentially simplifying the actual dynamic transmission processes. Our future work should incorporate multi factor (human factors) interactions, the implementation of early diagnosis (serological testing, population ultrasound screening) and targeted control measures (regular deworming of dogs, control of wildlife hosts, and health education) [63,64]. And the development of dynamic models to more comprehensively elucidates the transmission mechanisms of *Echinococcus* spp..
